# Left atrial volume and function are predictive of cardiac death or appropriate device therapy in patients with non-ischemic dilated cardiomyopathy

**DOI:** 10.1186/1532-429X-18-S1-Q21

**Published:** 2016-01-27

**Authors:** Punitha Arasaratnam, Archa Rajagopalan, Yoko Mikami, Khokan C Sikdar, Naeem Merchant, Jacqueline McQuaker, Andrew G Howarth, Bobby Heydari, James A White, Carmen P Lydell

**Affiliations:** 1 Stephenson Cardiac Imaging Centre, Libin Cardiovascular Institute of Alberta, Calgary, AB Canada; 2 grid.22072.350000000419367697Biostatistics, University of Calgary, Calgary, AB Canada

## Background

Several small studies have suggested that left atrial end-diastolic volume (LAEDV) is associated with adverse outcomes in patients with non-ischemic dilated cardiomyopathy (NICM). However, left atrial function, assessed through either LA end-systolic volume (LAESV) or the LA ejection fraction (LAEF) may provide incremental predictive utility. We sought to evaluate the prognostic value of LA volume and LAEF by Cardiovascular Magnetic Resonance (CMR) in a cohort of patients with NICM.

## Methods

117 patients (58% male) with NICM clinically referred for CMR were studied. Long axis cine images of the 2 and 4 chamber view were used to determine LAESV and LAEDV by a blinded reader using the area-length method. Values were indexed to body surface area using the Mostellar formula. Calculation of left ventricular (LV) volumes and EF were performed and the presence of any LV late gadolinium enhancement (LGE) was scored by an independent reader. All patients were followed for the composite outcome of cardiac death or appropriate implantable cardiac defibrillator (ICD) therapy. Cox proportional hazards models and Kaplan-Meier analysis were used to examine associations between LA measurements and the composite outcome.

## Results

Mean age was 57.1 ± 14.0 years and mean LVEF 31.8 ± 12.1%. Over a median follow-up of 689 days, 19 patients developed the composite outcome. In univariable analysis, ICD implantation, presence of LGE, LVEF, LAEDV, LAESV and LAEF were significantly associated with the composite outcome. Following adjustment for ICD implantation, hazard ratios (HRs) for each of the LA measurements were; LAEDVi: 1.32 per 10 ml/m^2^ (95%CI 1.05-1.67, p = 0.02), LAESVi: 1.52 per 10 ml/m^2^ (95%CI 1.21-1.91, p < 0.01), and LAEF: 0.66 per 10% (95%CI 0.50-0.87, p < 0.01). These measures remained independently associated with the composite outcome following adjustment for ICD implantation and LVEF with HRs of 1.33 (p = 0.01), 1.51 (p < 0.01), and 0.66 (p < 0.01), respectively. A multivariable model that replaced LVEF with presence of LGE as an independent variable showed similar results with HRs of 1.30 (p = 0.04), 1.47 (p < 0.01), and 0.68 (p = 0.01), respectively. Kaplan-Meier analysis showed only LAEF to have significant predictive value for event free survival (p = 0.04).

## Conclusions

Both LA volume and LAEF are associated with the occurence of cardiac death or appropriate ICD therapy among patients with NICM. However, LAEF is superior for the discrimination of event free survival and may therefore be a preferred measure of risk in this population.

**Figure 1:**
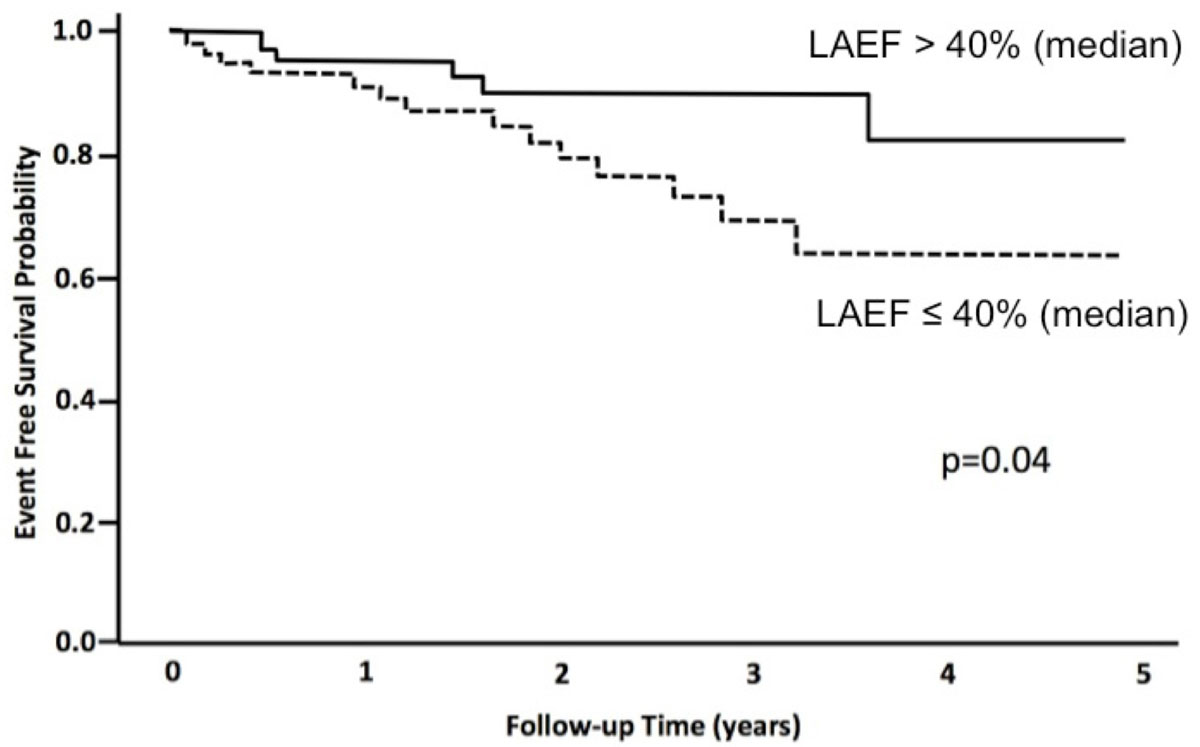
**Event free survival among NICM patients with LAEF above and below 40%**.

